# Cytotoxic Activity of Peripheral Blood Mononuclear Leukocytes, Activated by Interleukin-2/**β**-Cyclodextrin Nanocomposition against Androgen Receptor-Negative Prostate Cancers

**DOI:** 10.5402/2011/405656

**Published:** 2011-08-17

**Authors:** Natalia Yu. Anisimova, Andrey V. Sosnov, Nadezhda E. Ustyuzhanina, Gianfranco Baronzio, Mikhail V. Kiselevsky

**Affiliations:** ^1^N.N. Blokhin Russian Cancer Research Center, Russian Academy of Medical Sciences, Kashirskoe Shosse, 24, Moscow 115478, Russia; ^2^Chemical Diversity Research Institute, Rabochaya Street 2a, Khimki, Moscow 141401, Russia; ^3^N.D. Zelinsky Institute of Organic Chemistry, Russian Academy of Sciences, Leninsky Prospect 47, Moscow 119991, Russia; ^4^Family Medicine Area, ASL1 Legnano and “Metabloc Cancer Center,” c/o Centro Medico Kines, Castano Primo, 20022 Milan, Italy

## Abstract

Nanocomposition comprised of interleukin-2 in suboptimal noneffective concentration and **β**-cyclodextrin was studied *in vitro*. This preparation as well as interleukin-2 in optimal concentration was shown to increase natural killer activity to K-562 cells and cytotoxicity of activated peripheral blood mononuclear cells (PBMCs) against PC-3 and DU 145 cells. At the same time **β**-cyclodextrin or interleukin-2 in equimolar concentrations did not influence the spontaneous killer activity of PBMC. This combination of cyclodextrin + interleukin-2 led to the decrease of interleukin-2 effective concentration by an order. This phenomenon could be explained by cyclodextrins ability to promote the formation of nanoparticles with drugs, which results in enhancing their water solubility and bioavailability. Besides, interleukine-2/**β**-cyclodextrin nanocomposition as opposed to interleukin-2 alone led to increasing the number of not only lymphocytes, but also macrophages contained in activated PBMC population. Application of low concentration of interleukin-2 allowing for good clinical efficiency may significantly mitigate the side effects of the drug and enable to develop adoption of immunotherapy for patients with androgen-resistant prostate cancer.

## 1. Introduction

Prostate cancer is one of the most leading causes of death in men. In 2008 in the USA 286660 new deaths for prostate cancer were registered [1]. Prostate cancer accounts for about 12% of male deaths of cancer in the UK [2] and 10% in the USA [1] and is the second most leading cause of cancer death in men, after lung cancer. The majority of prostate cancer deaths (93%) occur in men aged 65 and over. In elder men aged 85 and over, prostate cancer overtakes lung cancer to become the most common cause of cancer deaths [3]. Two-stage development is characteristic for such kind of tumor, an androgen-dependent stage, when antiandrogen therapy is still effective, and androgen-resistant stage, when antiandrogen therapy does not work. The period of positive response towards antiandrogen therapy is limited and as a result most of the tumors enter the androgen-therapy-resistant stage. This significantly restricts therapeutic inventory and thus results in disappointing prognosis for patients.

Until recent times hormone-resistant prostate cancer has been considered incurable, and main therapeutic efforts have been applied towards life quality improvement for the patients affected by painful bone metastases. Therefore, over recent years prostate cancer biotherapy methods have been developed. Clinical trials with immune active agents reported in the last years have demonstrated efficacy in the treatment of prostate cancer. These agents include immune modulators, antibody targeting prostate-specific membrane antigen, and vaccines such as those targeting prostatic acid phosphatase, prostate-specific antigen, and cellular vaccines expressing granulocyte-macrophage colony stimulating factor [4–6]. Results from several recent clinical trials have suggested that immune-based therapies have clinical benefit in patients with prostate cancer with potentially less toxicity than traditional treatments [7–9].

Immunotherapy mediated by cytotoxic lymphocytes and lymphokine-activated lymphocytes (LAKs) was shown to be powerful in the destruction of drug-resistant tumor cells. Usually LAK generation requires recombinant interleukin-2 (IL-2). These lymphocytes kill target cells by two main mechanisms, namely the perforin pathway and the Fas-ligand (Fas-L) pathway. The androgen-independent prostate cells (AIPC) PC-3 and DU 145 express Fas on the cell surface but they are resistant to killing by anti-Fas antibody. AIPC is sensitive to killing by LAK, and the cell killing was primarily mediated through the Ca(2+)-dependent perforin pathway [10]. Since IL-2/LAK therapy has not proved to be clinically effective, improvement in efficiency and mitigation of systemic side effects are warranted. This improvement implies the development and the introduction of mononuclear leukocytes (MLs) activated by *exogenous cytokines*, or new methods of their cultivation. For this purpose liposomal and pegylated drug forms of interleukin-2 have been synthesized [11, 12].

Cyclodextrins, natural or synthetic *polysaccharides*, as nanocarriers for bioactive compounds offer new opportunities [13–15]. Thus, MLs of C3H/He mice activated by mixture of IL-2 and *β*-cyclodextrin-benzaldehyde possessed higher antitumor cytotoxic activity as compared to MLs activated only by IL-2 or *β*-cyclodextrin-benzaldehyde [16]. In this paper the influence of nanocomposition based on IL-2 in suboptimal concentration and *β*-cyclodextrin (*β*-CD) on immunophenotype and cytotoxic activity of ML towards prostate cancer cells of DU 145 and PC-3 types, as well as towards NK-sensitive K-562 cell, was studied.

## 2. Materials and Methods

### 2.1. Preparation of IL-2/*β*-CD Nanocomposition

Equal volumes of IL-2 solution “Proleukin” (Chiron, The netherPands) at the concentration level of 1000 IU in CM and *β*-CD (Sigma, USA) at the concentration level of 2 × 10^−4^ M in dimethylsulfoxide (DMSO) were mixed. The mixture was incubated at 30°C with the use of ST-3L shaker (ELMI, Latvia) under 800–1200 rpm for 45–60 min. The final concentrations of components in nanocomposition were 500 IU/mL for IL-2, 1 × 10^−4^ M for *β*-CD, and 50% DMSO in CM.

### 2.2. Cell Generation

#### 2.2.1. ML Extraction

ML was extracted from heparin stabilized periphery blood of healthy donor at ficoll-urografin gradient 1.077 g/cm^3^ (Sigma, USA) with the use of centrifugation under 400 g during 20–30 minutes. Lymphoid cells forming interphase ring were collected and washed out three times with RPMI-1640 medium (Sigma, USA). Cells were concentrated by centrifugation, calculated with the help of 5% trypan blue solution (PanEco, Russia), and then resuspended in complete medium (CM). CM is RPMI-1640 containing 10% of fetal bovine serum (HyClone Laboratories, Logan, UK) inactivated at 56°C for 30 min, 2 mM L-glutamine, 100 *μ*g/mL of penicillin and 100 ug/mL of streptomycin sulfate (PanEco, Russia). The concentration of the cells in suspension was equal to 5 × 10^5^-6 × 10^5^ cell/mL.

#### 2.2.2. LAK (IL-2^500^ Cells) Generation

The suspension of ML (5 mL) was mixed with IL-2 solution at the concentration level of 1000 U in CM (5 mL). The active concentration of IL-2 in the solution was 500 IU/mL. Cells were incubated in plastic flacks of 100 mL volume in CO_2_ incubator for 48 h at 37°C in 5% CO_2_ atmosphere.

#### 2.2.3. IL-2^50^ Cells Generation

The suspension of ML (9.5 mL) was mixed with IL-2 solution at the concentration level of 1000 IU in CM (0.5 mL). The active concentration of IL-2 in the solution was 50 IU/mL. Cells were incubated in plastic flacks of 100 mL volume in CO_2_ incubator for 48 h at 37°C in 5% CO_2_ atmosphere.

#### 2.2.4. IL-2^50^/*β*-CD Cells Generation

The suspension of ML (4.5 mL) was mixed with IL-2/*β*-CD composition (0.5 mL). The active concentrations of components in the solution were 50 IU/mL for IL-2, 1 × 10^−5^ M for *β*-CD, and 5% for DMSO. Cells were incubated in plastic flacks of 100 mL volume (Costar, USA) in CO_2_-incubator (Guan, France) for 48 h at 37°C in 5% CO_2_ atmosphere.

#### 2.2.5. *β*-CD-Cells Generation

The suspension of ML (4.5 mL) was mixed with the solution of *β*-CD at the concentration level of 1 × 10^−4^ M in DMSO (0.5 mL). The active concentration of *β*-CD in the solution was 1 × 10^−5^ M. Cells were incubated in plastic flacks of 100 mL volume in CO_2_-incubator for 48 h at 37°C in 5% CO_2_ atmosphere.

### 2.3. Antitumor Activity Assay

NK activity was determined towards NK-sensitive human erythroleukemia cell line K-562. Cytotoxic activity was estimated towards PC-3 and DU 145 AIPC cell lines. Tumor cells (1 × 10^4^ in 1 mL CM) were incubated with IL-2^50^/CD cells, LAK (IL-2^500^ cells), IL-2^50^-cells, *β*-CD cells and control ML at a ratio of 1 : 10 (K-562) or 1:2 (PC-3 and DU 145) for 24 h at 37°C in 5% CO_2_ atmosphere. MLs incubated with 5% DMSO were used as a control. Cells were controlled and documented by Axiovert (Zess, Germany). We added 20 *μ*L Thiazolyl Blue Tetrazolium Bromide (MTT) (Sigma, USA) 5 g*·*L^−1^ to each well of multiwell culture plate, incubated for 4 hours, then added 150 *μ*L DMSO, mixed about 10 min until the crystal completely dissolved. 

 Index of cytotoxic activity (IC) was calculated from optical density value measured by tray photometer (Multiscan MS, Finland) after addition of Thiazolyl Blue Tetrazolium Bromide (MTT) (Sigma, USA). IC, % = (1−(OP_CC+ML_−OP_ML_)/OP_CC_) × 100—OP_CC+ML_—optical density in the vessel containing cancer cells and ML, OP_ML_—optical density in the vessel containing ML only, OP_CC_ —optical density in the vessel containing cancer cells only.

### 2.4. Flow Cytometric Analysis

The cells were washed by PBS and incubated with antihuman antibodies for 30 min at 4°C. MLs were incubated with monoclonal antibodies specific for human antigens CD. Antibodies used were anti-CD3, anti-CD4, anti-CD16, anti-CD25, anti-CD56, anti-CD58, anti-CD80, anti-CD11c, anti-CD83, and anti-HLA-DR (Beckman Coulter, USA). Flow cytometric analysis was done using a Coulter FC500 flow cytometer (Coulter, USA).

### 2.5. Statistical Analysis

CellQuest and WinMDI.2.8. software were used for statistical analysis of cell phenotype. All statistical calculations were made using the software package Statistica 6.0 using factor dispersion analysis (ANOVA/MANOVA) and Mann-Whitney *U* test for group comparison. In all cases, results were considered as statistically significant at *P* < 0.05.

## 3. Results

### 3.1. NK Activity of Activated Cells

**Figure 1:**
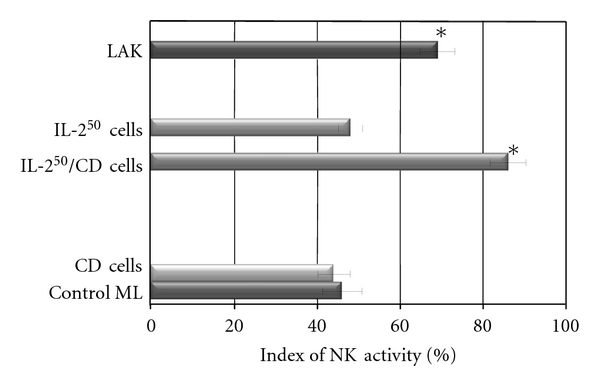
NK activity of IL-2^50^/CD cells versus control ML, LAK, CD cells, and IL-2^50^ cells (IL-2 cells (50 U/mL)), *P < 0.05.

**Figure 2:**
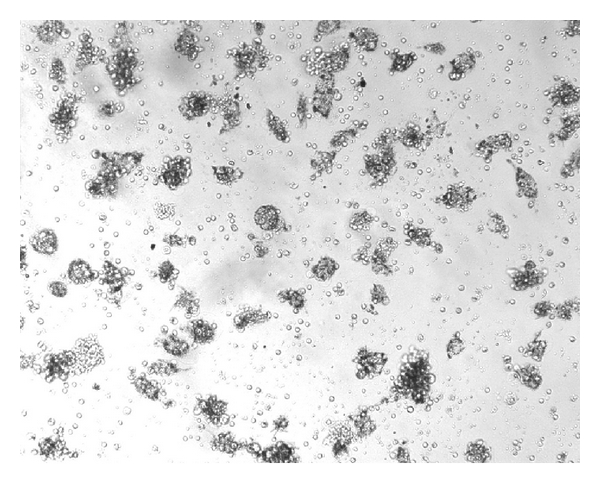
Microphoto of LAK after 18 h coincubation with K-562 (×100). MTT added.

**Figure 3:**
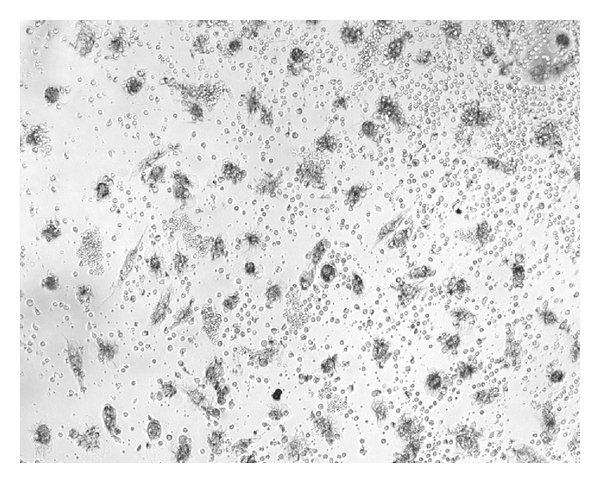
Microphoto of IL-2^50^/CD cells after 18 h coincubation with K-562 (×100). MTT added.

**Figure 4:**
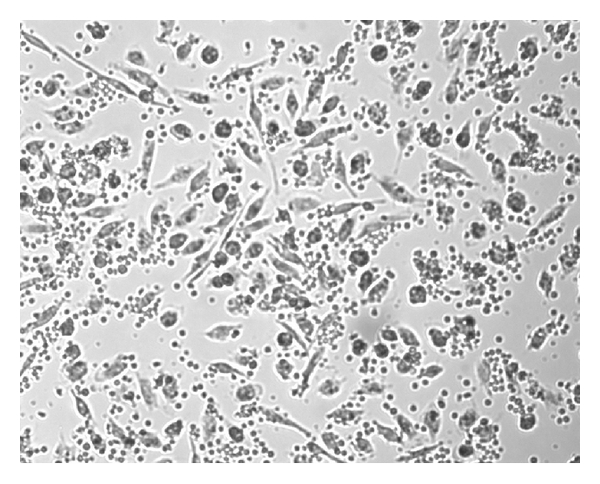
Microphoto of IL-2^50^ cells after 18 h coincubation with K-562 (×100). MTT added.

**Figure 5:**
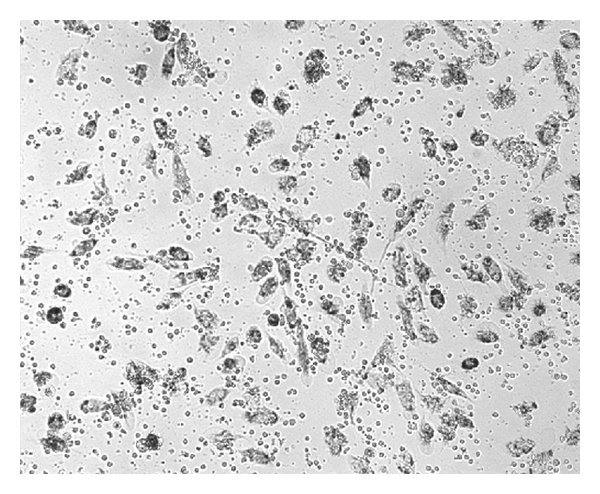
Microphoto of control ML after 18 h. coincubation with K-562 (×100). MTT added.

**Figure 6:**
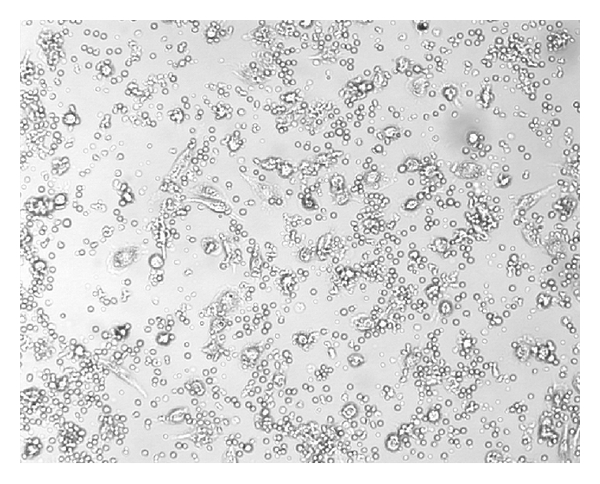
Microfoto of control ML after 18 h. coincubation with K-562 (×100). MTT added.

NK activity of IL-2^50^/*β*-CD cells, LAK, IL-2^50^ cells, *β*-CD cells, and control ML was detected at NK-sensitive cells line K-562. The cancer cell/effector cell ratio was 1:10. LAK generated with high concentration of IL-2 activated lysis of 67 ± 10, 1%  K-562 cells as compared to control ML (IC = 46 ± 7.8%). IL-2^50^/*β*-CD cells activated by nanocomposition containing suboptimal concentration of IL-2 demonstrated higher cytotoxic activity (86 ± 7.2%), as compared to LAK (Figure 1). ML incubation with *β*-CD as well as with low concentration of IL-2 did not lead to cell killer activity stimulation. As microphoto showed the IL-2^50^/*β*-CD cells population as well as LAK contained a large number of lymphocyte clusters that indicated the proliferation of these cells (Figures 2 and 3). On the contrary IL-2^50^ cells had not organized such clusters (Figure 4). Unlike LAK and control ML (Figure 5) the IL-2^50^/*β*-CD composition results in the generation of a large number of adherent dendrite-like cells. Obviously this effect is caused by the activity of *β*-CD, which alone initiates the appearance of similar cells (Figure 6).

### 3.2. Flow Cytometry Analysis

**Figure 7:**
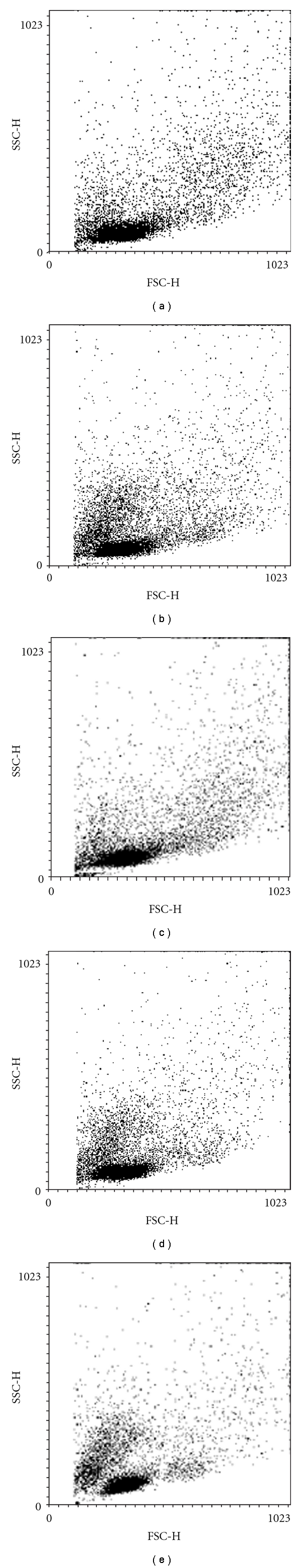
Induction of new cell populations of activated peripheral blood mononuclear cells versus control (SSC-H:site scatter height, FSC-H:forward scatter height). (a) control ML, (b) LAK, (c) IL-2^50^ cells, (d) CD cells, and (e) IL-2^50^/CD cells.

The analysis revealed that the IL-2^50^/*β*-CD cells population as opposed to control cells has two cell regions (Figures 7(a), 7(b), 7(c), 7(d), and 7(e)). The second additional IL-2^50^/*β*-CD cells region is obviously represented by large macrophage cells. IL-2^50^/*β*-CD cell culture as opposed to IL-2^50^ cell is represented by adherent cells, which is not typical for LAK. In particular, increasing the expression of NK markers (CD16/CD56), NK-T cells markers (CD3/CD16), as well as adhesion molecules (CD58), and receptor cell towards IL-2 (CD25) was detected (Table 1). However, as compared to LAK, the population of IL-2^50^/*β*-CD cells contained a large number of antigen-presenting, mature CD11c+ dendritic cells, which express HLA-DR and CD80/CD83 cell markers represented mainly in region 2.

### 3.3. Cytotoxic Activity of Cells Tested towards AIPC Cell Lines

**Figure 8:**
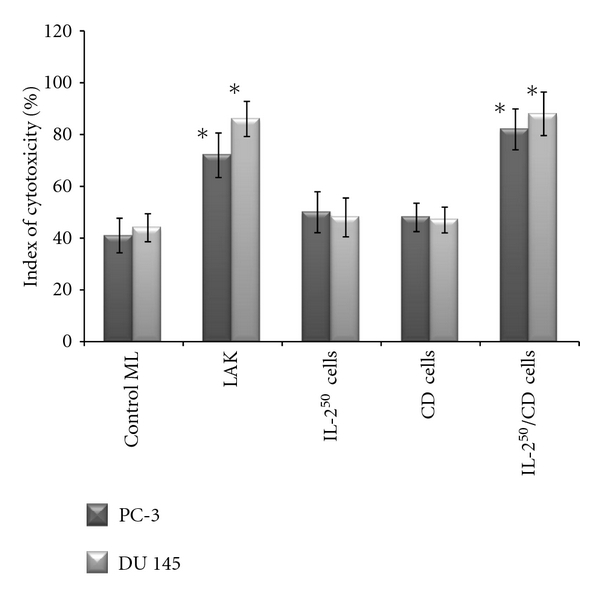
Cytotoxic activity of activated cells as compared to control ML towards AIPC prostate cancer cell lines PC-3 and DU 145, *P < 0.05.

According to data presented in Figure 8 spontaneous killer activity of control ML was equal to 41 ± 5.2% and 44 ± 8.0% for PC-3 and DU 145 cells, respectively. IL-2^50^ cells and *β*-CD cells had almost the same activity as control ML (there was no proved distinction revealed). LAK possessed better cytotoxic potential and on the average lysed 72 ± 5.1% and 86 ± 8.2% PC-3 and DU 145 cells, respectively. IL-2^50^/*β*-CD cells were slightly more active against PC-3 and DU 145 cells, (82 ± 5.2% and 88 ± 10.2%, resp.), as compared to LAK, but that difference was not proved statistically.

## 4. Discussion

At present a therapy of hormone-resistant prostate cancer possesses very limited resources. In addition, modern methods of chemotherapy and X-ray therapy often result in side effects, including immunosuppression and thus impair patient's life quality. The undermining of immunological and antitumor status as a result of the treating may lead to the disease progression. Therefore, it seems to be of current interest to develop new methods of biotherapy of hormone-resistant prostate cancer. At present different types of dendritic-cells-based vaccine, are undergoing clinical trials [17, 18]. IL-2/LAK-immunotherapy can be considered to be one of the top priority issues. This is proved by experimental investigations which reveled high sensitivity of human AIPC cells to cytotoxic activity of LAK [3].

As mice experiments have previously shown, the combination of IL-2 and *β*-cyclodextrin-benzoic acid enables to improve the LAK killer activity towards sarcoma cells [9]. It was proved that *β*-CD composition with suboptimal noneffective concentration of IL-2 improves NK-activity, tested on K-562, as well as cytotoxicity of ML against PC-3 and DU 145. In this case equimolar concentration of *β*-CD itself did not produce any significant effect upon spontaneous killer activity of ML. This phenomenon can be explained by the well-known ability of *β*-CD to improve water solubility and biological availability of drugs, including IL-2 [13, 15]. Besides, unlike IL-2 itself, IL-2/*β*-CD nanocomposition leads to increase in the number of not only lymphocytes but also of macrophages/dendritic cells in ML population as well. It is well-known that macrophages and especially dendritic cells (DCs) secrete cytokines, particularly IL-12, thus improving IL-2 effect. It is worth mentioning that IL-2^50^/*β*-CD cells population contains mature CD80+/CD83+ dendritic cells, which express molecules of antigen presentation HLA-DR. This can also prove the ability of IL-2/*β*-CD nanocomposition to activate effectors of natural and adaptive immunity.

The main disadvantage of the current adoptive immunotherapy methods is the limited target effect produced upon one of the mentioned immune system components. In particular, DC vaccines action is aimed to the creation of specific immune response to the tumor antigen. However, as tumor is heterogenic, a part of the cells population does not express cancer-associated antigens and MHC molecules enabling so the neoplastic cells to escape an immune response effect. In contrast, LAK cells lyse cancer cells by antigen and MHC-independent way [19]. MHC expression on cancer cells downregulates NK and LAK activity [20, 21].

Application of IL-2^50^/*β*-CD nanocomposition was suggested to eliminate the above-mentioned methods drawbacks because *it* provides a unique origin for LAK and DC. Usually immune clinical cancer treatment involves the use of high cytokine dose, reaching in the case of IL-2 dose near or superior to 18000000 IU. This dose creates serious side effects [22]. The data we have obtained proves that IL-2/*β*-CD nanocomposition enables to decrease efficient concentration of IL-2 at least by one order of magnitude. Hence, creation of nanocapsules based on *β*-CD and cytokines may significantly improve adoptive immunotherapy for cancer patients. The potential of *β*-CD in drug delivery applications remains partially unexploited [23]. We have proved tested nanocomposition to be highly efficient towards AIPC cells. Therefore further detailed preclinical studies aimed at creating new effective drugs and methods of immunotherapy of patients with hormone-resistant prostate cancer are worth being carried out.

## Figures and Tables

**Table 1:** Phenotype of IL-2^50^/CD cells, CD cells, IL-2^50^ cells, and LAK versus control ML.

Surface cell markers	Control ML	LAK	IL-2^50^ cells	CD cells	IL-2^50^/CD ML
CD3/CD16	5.86	16.56*	11.82	6.55	22.3*
CD16	5.22	6.22	10.56	5.21	8.3
CD4/CD25	13.05	21.76*	18.82	11.16	19.14*
CD25	2.58	5.21	1.8	4.7	7.8*
CD58	4.18	29.32*	17.56	30.64*	34.45*
HLA-DR	3.26	3.98	1.68	5.71*	5.22*
CD80	0.57	0.78	0.47	0.14	0.26
CD80/CD83	9.81	12.42	9.18	11.25	14*
CD83	2.89	3	3	4.43	2.59
CD11c	8	9.91	8.53	14.11	13.44*
CD56/CD16	7.82	9.1	5.1	0	12.3*

*Significant date versus control ML (*P* < 0.05).
